# Sequencing and analysis of the complete mitochondrial genome of *Ochotona hyperborea* from China and its phylogenetic analysis

**DOI:** 10.1080/23802359.2021.1934137

**Published:** 2021-05-31

**Authors:** Xin-Xu Zhao, Zhong-Wan Piao, Qiao-Jiang Yang, Qi Zhang, Qian-Qian Yao, Jun-Sheng Zhang, Zhu Liu

**Affiliations:** College of Life Science and Technology, Mudanjiang Normal University, Mudanjiang, PR China

**Keywords:** Control region, mitogenome, phylogenetic trees, *Ochotona hyperborea*

## Abstract

The complete mitogenome sequence of *Ochotona hyperborea* was determined using long PCR. The genome was 17,063 bp in length and contained 13 protein-coding genes, two ribosomal RNA genes, 22 transfer RNA genes, one origin of L strand replication, and one control region. The overall base composition of the heavy strand is A (31.1%), C (28.7%), T (26.3%), and G (13.9%). The base compositions present clearly the A–T skew, which is most obvious in the control region and protein-coding genes. Mitochondrial genome analyses based on MP, ML, NJ, and Bayesian analyses yielded identical phylogenetic trees. This study verifies the evolutionary status of *Ochotona hyperborea* in Ochotonidae at the molecular level. The mitochondrial genome would be a significant supplement for the *Ochotona hyperborea* genetic background. The eight *Ochotona* species formed a monophyletic group with the high bootstrap value (100%) in all examinations.

The distribution of *Ochotona hyperborea* Pallas, 1811 is a typical species distributed in Paleobe (Lissovsky 2005). The distribution area of the *Ochotona hyperborea* is extending eastward from the Ural Mountains to Sakhalin Island, Kamchatka Peninsula and Hokkaido, Japan (Lissovsky et al. 2007). The distribution area in China is northeast of China. In China, the species was considered to include two subspecies (*O. h. coreana* and *O. h. hyperborea*). The *O. h. coreana* distributed in the Changbai Mountains is currently considered to be an independent species (*O. coreana*) (Lissovsky 2014; Liu et al. 2017). The sample in this study is from typical *O. h. hyperborea* distributed areas, including Daxinganling and Xiaoxinganling (Liu et al. 2017).

A muscle sample was obtained from a female *Ochotona hyperborea* captured from the Yichun regions of Xiaoxinganling Mountains in Heilongjiang Province, China (48°12′54″N, 129°25′14″E). The muscle tissue was preserved in 95% ethanol and stored at −75 °C before use. The specimen and its DNA is stored in Animal and Plant Herbarium of Mudanjiang Normal University. The voucher number is DBST2019004. Genomic DNA was extracted from muscle using the EasyPure genomic DNA kit (TransGen Biotech Co., Beijing, China). The 14 pairs of primers used in PCR were designed based on the existing *Ochotona* mitochondrial genome. The sequencing of the sequence is carried out using the first-generation sequencing technology and the ABI 3730 sequencer (Ruiboxingke Biotechnology Co. Ltd., Beijing, China). The draft sequence was manually spliced and corrected. The complete mitochondrial genome sequence was annotated using Sequin.

The mitochondrial genome is a circular double-stranded DNA sequence that is 17,063 bp long including 13 protein-coding genes, two rRNA genes, 22 tRNA genes, one origin of L strand replication, and one control region. The accurate annotated mitochondrial genome sequence was submitted to GenBank with accession number MW270995. The arrangement of the multiple genes is in line with other Lagomorpha species (Wang and Yang 2012; Yu et al. 2015; Ding, Chen, Pan, et al. 2016; Ding, Chen, Wang, et al. 2016; Giannoulis et al. 2018; He et al. 2018; Yang et al. 2019; Zhang et al. 2020) and most mammals (Mouchaty et al. 2000; Nikaido et al. 2001, 2003; Fontanillas et al. 2004; Cabria et al. 2006; Meganathan et al. 2012; Xu et al. 2012, 2013; Kim et al. 2013, 2017; Yoon et al. 2013; Huang et al. 2014, 2016; Hou et al. 2016; Liu et al. 2016; Xu et al. 2016; Jin et al. 2017; Liu, Tian, Jin, Dong, et al. 2017; Liu, Tian, Jin, Jin, et al. 2017; Liu, Wang, et al. 2017; Gutiérrez et al. 2018; Jia et al. 2018; Liu et al. 2018; Liu, Dang, et al. 2019; Liu, Qin, et al. 2019).

The control region of *Ochotona hyperborea* mitochondrial genome was located between the tRNA-Pro and tRNA-Phe genes, and contains only promoters and regulatory sequences for replication and transcription, but no structural genes. Three domains were defined in *Ochotona hyperborea* mitochondrial genome control region (Zhang et al. 2009): the extended termination-associated sequence (ETAS) domain, the central conserved domain (CD), and the conserved sequence block (CSB) domain.

The total length of the protein-coding gene sequences was 11,400 bp. Most protein-coding genes initiate with ATG except for ND2, ND3, and ND5, which began with ATC or ATT. Eight protein-coding genes terminated with TAA. The incomplete stop codons (T––) were used in COX3, ND6, ND3, and ND4. A strong bias against A at the third codon position was observed in the protein-coding genes. The frequencies of CTA (Leu), ATT (Ile), TTA (Leu), and ATA (Met) were higher than those of other codons. The length of tRNA genes varied from 59 to 75 bp.

Most *Ochotona hyperborea* mitochondrial genes were encoded on the H strand, except for the ND6 gene and eight tRNA genes, which were encoded on the L strand. Some reading frame intervals and overlaps were found. One of the most typical was between ATP8 and ATP6. The L-strand replication origin (OL) was 31 bp long and had the potential to fold into a stable stem-loop secondary structure. The total base composition of *Ochotona hyperborea* mitochondrial genome was A (31.1%), C (28.7%), T (26.3%), and G (13.9%). The base compositions clearly present the A–T skew, which was most obviously in the control region and protein-coding genes.

In order to explore the evolution of Lagomorpha species which include Ochotonidae and Leporidae, especially the evolution of genus *Ochotona* from China; here, we investigate the molecular phylogenetics of Chinese *Ochotona hyperborea* using complete mitochondrial genome sequence of 22 species. All sequences generated in this study have been deposited in the GenBank (Figure 1).

**Figure 1.**
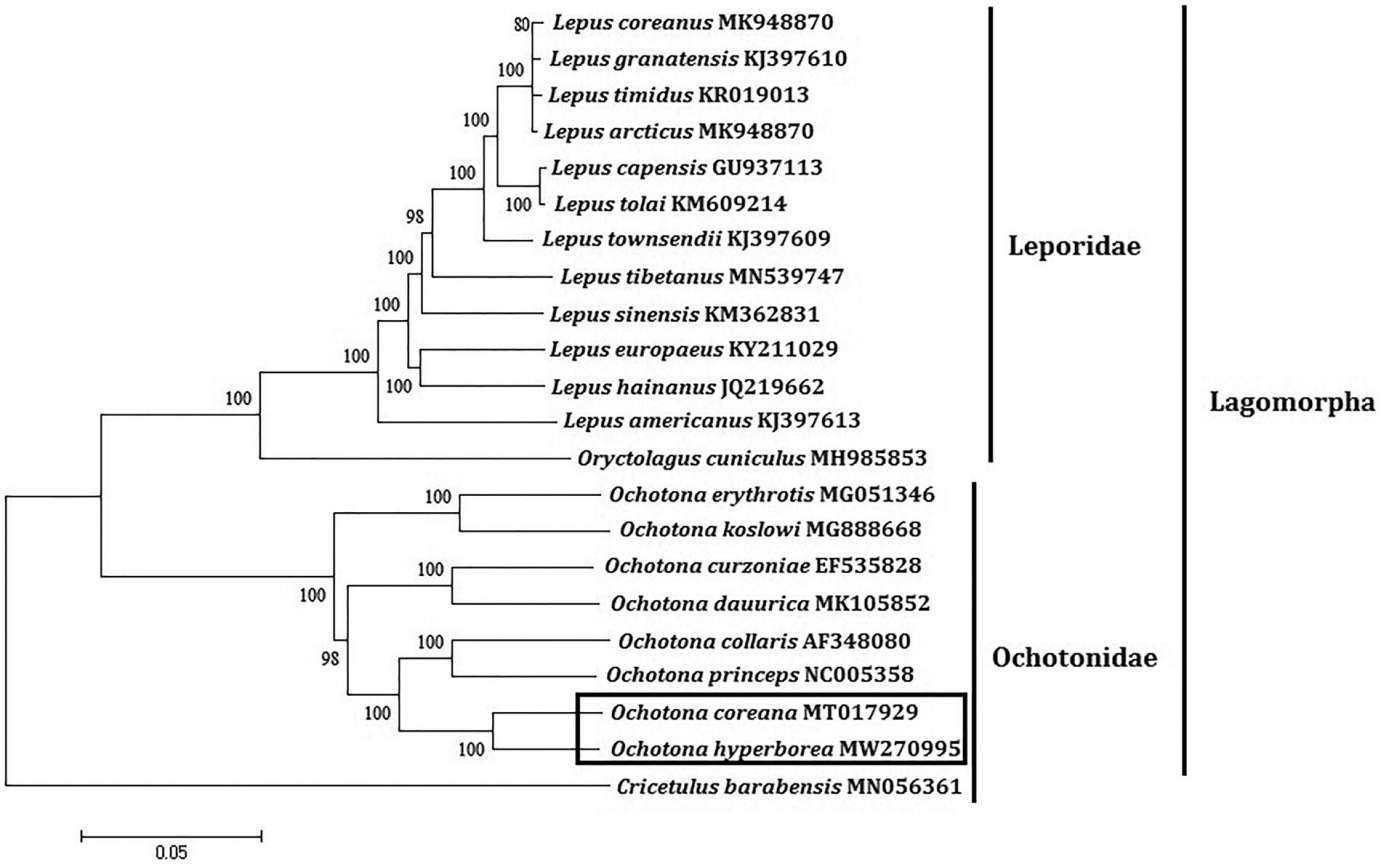
Phylogenetic tree generated using the maximum-likelihood method based on complete mitochondrial genomes. The outgroup is *Cricetulus barabensis* (MN056361).

Mitochondrial genome analyses based on MP, ML, NJ, and Bayesian analyses yielded identical phylogenetic trees, indicating a close phylogenetic affinity of species. The phylogram obtained from ML method is shown in Figure 1. It shows that two major phyletic lineages were present in Lagomorpha: Ochotonidae and Leporidae. Ochotonidae comprised *Ochotona hyperborea*, *Ochotona coreana*, *Ochotona dauurica*, *Ochotona erythrotis*, *Ochotona curzoniae*, *Ochotona koslowi*, *Ochotona collaris*, and *Ochotona princeps* was supported by bootstrap values of 100%. Leporidae comprised *Lepus arcticus*, *Lepus hainanus*, *Lepus timidus*, *Lepus coreanus*, *Lepus tolai*, *Lepus sinensis*, *Lepus tibetanus*, *Lepus europaeus*, *Lepus capensis*, *Lepus americanus*, *Lepus granatensis*, *Lepus townsendii*, and *Oryctolagus cuniculus* was supported by bootstrap values of 100%. This study verifies the evolutionary status of *Ochotona hyperborea* in Ochotonidae at the molecular level. The mitochondrial genome would be a significant supplement for the *Ochotona hyperborea* genetic background. The eight *Ochotona* species formed a monophyletic group with the high bootstrap value (100%) in all examinations.

## Data Availability

Mitogenome data supporting this study are openly available in GenBank at https://www.ncbi.nlm.nih.gov/nuccore/MW270995, Associated BioProject, https://www.ncbi.nlm.nih.gov/bioproject/PRJNA721816, and BioSample accession number at https://www.ncbi.nlm.nih.gov/biosample/SAMN18739897.
